# Fighting Zoom fatigue: Evidence-based approaches in university online education

**DOI:** 10.1038/s41598-025-90973-6

**Published:** 2025-02-27

**Authors:** Johannes M. Basch, Patrick Albus, Tina Seufert

**Affiliations:** 1 Hochschule Neu-Ulm, Neu-Ulm, Germany; 2 Institute of Psychology and Education, Department for Learning and Instruction, Ulm University, Lise-Meitner Straße 16, 89081 Ulm, Germany

**Keywords:** Zoom fatigue, Videoconference fatigue, Online teaching, Online education, Human behaviour, Statistics

## Abstract

As the world has rapidly transitioned to virtual platforms for education due to the Covid-19 pandemic, a new phenomenon known as “Zoom fatigue” has emerged. This term refers to the exhaustion associated with the extensive use of virtual communication platforms. In this context, the present study investigates the effects of different virtual class settings on cognitive load and fatigue. Four interventions were examined: self-view on vs. self-view off, focus-view function vs. grid-view function, activating a virtual background vs. natural background, and active participation vs. no active participation. The results suggest that turning off self-view can significantly reduce both cognitive load and fatigue. However, the effects of focus-view function vs. grid-view function did not reach significance for either cognitive load or fatigue. Similarly, using a virtual background vs. a natural background did not significantly affect fatigue, but it did influence cognitive load, with higher values for virtual backgrounds. Lastly, active participation in classes was associated with decreased fatigue and lower extraneous cognitive load compared to no active participation. The findings highlight the importance of considering both technological aspects (e.g., self-view, background settings) and pedagogical approaches (e.g., active participation) in online teaching to promote a more engaging and less fatiguing learning environment.

## Introduction

Online teaching formats have recently gained importance due to increasing digitalization. The Covid-19 crisis has also meant that online teaching formats have suddenly become not only alternatives to face-to-face teaching, but in some cases the only way for teachers and university lecturers to impart knowledge to students.

However, while many have come to appreciate the flexibility of teaching and learning from the comfort of their own four walls, the phenomenon of “Zoom fatigue” (hereafter we use the term “videoconference fatigue” as a proxy for all videoconferencing programs) has become prevalent in organizational meetings as well as among students and teachers. Videoconference fatigue is defined as the phenomenon of feeling “exhausted or tired attributed to a videoconference”^1^^, p. 330^.

Although there is some information available on the causes of videoconference fatigue and many suggestions on how to avoid it [e.g.,^2,3^], there is little empirical research on how to combat videoconference fatigue and whether the various methods suggested in qualitative research or practitioner articles actually work. Finding empirically based methods to combat videoconference fatigue is important because technology-mediated instructional formats increased already before the Covid-19 pandemic, became prevalent during the pandemic, and are still used to a high extent after the pandemic. Furthermore, interventions can be applied not only to online teaching, but also to videoconference meetings in general. Therefore, drawing on media richness theory^4^, Cognitive Load Theory^5^, and several practical suggestions [e.g.,^6^], we tested four interventions in university teaching during the Covid-19 pandemic.

## Background

### Videoconference fatigue

As mentioned earlier, the phenomenon of videoconference fatigue refers to “the degree to which people feel exhausted, tired, or worn out as a result of videoconferencing”^1^^, p.331^. According to Bennett, et al.^1^ videoconference fatigue can be distinguished from “normal” and work fatigue such that videoconference fatigue is dependent on specific events (i.e., the videoconference) and demands rather than general work demands. Furthermore, videoconference fatigue differs from work fatigue in its temporal nature, because it is closer to the event (i.e., during and righter after the videoconference) rather than feeling exhausted at the end of a long work day. Videoconference fatigue can be accompanied by various symptoms, such as exhaustion, anxiety, concentration problems, headaches, back pain, depressed mood, insomnia, etc.^6–8^.

One of the areas where videoconferencing has been increasingly used in recent years has been in teaching. Prior to the pandemic, only 46% of lecturers in the US had already made experience with online courses^9^. This suddenly changed with the outbreak of the Covid-19 pandemic, which necessitated the adoption of online teaching as the sole means of ensuring both educational continuity and adherence to social distancing guidelines^10^. However, the use of videoconferencing systems in higher education is also associated with different learning experiences, teaching formats, and outcomes [see^11^, for an overview]. In contrast to the great flexibility associated with online teaching, Peper, et al.^6^ found that only 6% of the students preferred online learning to onsite learning, and 80% reported that attending classes via Zoom was significantly more difficult than attending face-to-face classes. Furthermore, the authors note that the lack of visual and nonverbal feedback, self-monitoring in videoconferencing settings, and the physical act of un-muting before interacting with others may be additional barriers to effective communication and, therefore, for a beneficial learning experience in videoconference teaching. Not only in education, but also in managerial contexts, studies have shown that videoconference fatigue is not due to meetings per se, but that videoconference meetings are perceived as more exhausting than regular meetings^12^. In a survey conducted by Rump and Brandt^13^, 77% of the participants reported that videoconference fatigue sometimes occurs, while 15% perceive videoconference fatigue as a constant stressor. This contrasts somewhat with the findings that videoconference meetings are perceived to be more effective^14^, less energy-draining^15^, and less time-consuming than regular meetings^16^. According to Bickmeyer^17^, videoconference meetings are more about sharing information than building mutual trust. Additionally, Nesher Shoshan and Wehrt^12^ found that meeting duration, meeting size, or a moderator of a meeting did not moderate the relationship between meeting format and exhaustion.

### Possible explanations for videoconference fatigue

In the course of research and experience in recent months and years, various explanations for the development of videoconference fatigue have emerged. They can be derived from different theoretical models and perspectives. This manuscript will start by examining the cognitive impact of videoconference fatigue on learners. Although there are other factors in play when it comes to learning online such as affective and social factors^18^, this research provides a necessary building block in exploring this new fatigue. Furthermore, we also need to consider communication aspects as well as the parallel reality behind the screen^6^.

From an information processing perspective, one can ask what participants have to process, i.e., what is the incoming information? This includes visual information, such as images of other participants, and auditory information, such as what’s being said. According to media richness theory^4^, videoconferencing provides almost the same level of media richness as face-to-face interactions because it uses the same channels of information transmission, i.e., verbal, nonverbal, and paraverbal information. However, the videoconference format is still less rich than face-to-face interactions because the image captured by a webcam represents only a small part of the actual reality. Therefore, nonverbal behavior is limited to this small image. Thus, one source of videoconference fatigue is that participants need to be more attentive and invest more resources to capture all the relevant nonverbal cues. Given other design considerations, such as the gallery or grid-view in videoconference programs, it might be particularly challenging to pay attention to the nonverbal behavior of many individuals at the same time and to split the limited attention^6^. Moreover, even if participants would attend all the relevant images, the information might be blurred or fragmented. In either case, the visual information would convey not only what is relevant, but also extraneous aspects that must be actively suppressed. Based on Cognitive Load Theory [CLT,^5^], these elements may contribute to extraneous cognitive load that is not directly related to the learning task. In addition, auditory information may serve as another source of extraneous cognitive load, as lag times could affect the fluency of the speech, thus affecting synchrony and ultimately the quality of communication^7,19–21^. Contrary to face-to-face meetings, the task of filtering out background noise from unmuted microphones becomes more complex, as the challenge in spatial localization hampers the ability to discern its relevance. These extraneous factors inherent in videoconferencing could potentially contribute to fatigue.

As mentioned above, videoconferences are not only about receiving and processing information, but rather are communicative situations in which signals are received but also sent beyond the video stream and verbalizations. A crucial aspect of communication is the gaze, which, according to Bailenson^22^, can cause nonverbal overload and thus also might contribute to videoconference fatigue. Compared to face-to-face interactions, it is rather uncomfortable and also unusual for us to maintain close eye contact for long periods of time. Given the grid-view, it is also not normal for us to have multiple people staring at us when we are not even the center of attention at that moment. What has often been mentioned as a possible explanation for videoconference fatigue, is constant self-mirroring^17^. Bailenson^22^ describes the difficulty of self-mirroring in videoconferences with the metaphor that one should imagine a colleague walking by with a mirror that shows one’s own face all day. A meta-analysis by Fejfar and Hoyle^23^ found a small effect of looking at oneself in a mirror and negative affect. Additionally, constant self-monitoring may lead to more impression management^24^. Additionally, not only can the lack of receiving nonverbal cues can be ambiguous^20^ and exhausting, but sending additional nonverbal cues to improve mutual understanding can consume additional resources that would not have been necessary for mutual understanding in a face-to-face context^22^.

A third perspective on the potential causes of videoconferencing fatigue is the off-screen environment, or the participant’s actual environment. This environment can serve as a source of both unavoidable and self-induced distractions. Often, participants have little control over distracting sounds or activities in their environment, yet these elements can still divert their attention. Moreover, participants themselves often create distractions by engaging in alternative tasks, such as checking email or browsing the Internet, facilitated by devices that are typically within easy reach^6,25^. Even household activities, such as cleaning or cooking, can interfere with attention^2^. These constant potential distractions provide a continuous temptation to divert attention away from the primary task.

These additional tasks, which are not directly relevant to the videoconferencing task, contribute to an increase in extraneous cognitive load, potentially leading to increased fatigue. Furthermore, participants may also need to allocate cognitive resources to aspects of the videoconference that are inherent but not directly relevant to the meeting, such as the constant gaze of many participants in the grid-view, the continuous self-mirroring, or the blurring of information by the virtual backgrounds of other participants. These affordances can further contribute to extraneous cognitive load. As mentioned above, these aspects of extraneous cognitive load can particularly lead to videoconferencing fatigue.

In contrast, videoconferences require participants to focus on the primary learning content. This requires close monitoring of a variety of communication cues, including visual, verbal, and nonverbal signals. Often, these cues must be tracked across multiple communication partners, as is the case in grid-views. This focus on primary learning content requires an investment of cognitive resources to process and integrate new information. This investment is critical for effective learning and comprehension because it directly contributes to the processing and integration of new information. In Cognitive Load Theory, this mental effort that is germane to the task itself is referred to as germane cognitive load^5^. Thus, germane cognitive load represents the cognitive resources that participants allocate to understanding the learning content of the videoconference.

However, it’s also important to consider intrinsic cognitive load. It refers to the mental effort and resources required to process and understand information due to its inherent complexity. The level of intrinsic cognitive load experienced is also influenced by the novelty of the information and the learner’s prior knowledge and expertise in the domain^5^. In the context of videoconferencing fatigue, some settings might influence the perceived difficulty of the learning material, particularly when certain presentation formats of the content are suboptimal, leading students to perceive the material as more challenging and consequently increasing the intrinsic cognitive load experienced by participants.

Therefore, when considering how to reduce videoconferencing fatigue, one needs to consider the balance between intrinsic, extraneous and germane cognitive load. One approach is to minimise extraneous cognitive load. Another approach is to increase germane cognitive load by helping to focus on relevant processes during videoconferencing. In our study, we investigate four different approaches, three of which primarily aim to reduce extraneous cognitive load, and one of which primarily aims to increase germane cognitive load.

### Possible interventions – development of hypotheses

There has been limited empirical research on strategies to mitigate videoconference fatigue. As previously discussed, this fatigue can stem from cognitive overload, which is often associated with extraneous processes. One potential intervention to reduce such extraneous demands could be to turn off one’s self-view. Not only being watched by others, but also looking at yourself on the screen for long periods of time, like in a mirror, can be draining. As suggested in several conceptual papers, turning off self-view could be a potential strategy to alleviate videoconference fatigue^7,22^. In terms of Cognitive Load Theory, disabling self-view could reduce extraneous cognitive load, potentially allowing participants to invest more cognitive resources, or germane cognitive load, into the conference, thereby enhancing their engagement and comprehension. However, there is hardly any empirical evidence supporting this proposition, both in terms of reducing extraneous cognitive load and mitigating videoconference fatigue. Based on the additivity hypothesis of cognitive load theory, it could also be postulated that the cognitive resources freed up by reducing extraneous cognitive load could be redirected to germane processes, thereby increasing germane cognitive load. Therefore, we assume:

**Hypothesis 1:** Disabling the self-view in videoconferences will result in lower levels of fatigue compared to when self-view is enabled. Furthermore, we anticipate a reduction in extraneous cognitive load and an increase in germane cognitive load when self-view is disabled.

As another possible intervention, we want to take a closer look at the difference between grid-view and focus-view. As mentioned above, according to Bickmeyer^17^, it is not normal to be stared at by many faces all the time. Moreover, in educational contexts, it can be very challenging for teachers to pay attention to all of the faces during grid-view^6^. Accordingly, grid-view results in a loss of group visualization compared to face-to-face interactions, because one cannot focus the whole group at once and must therefore pay attention to each webcam image separately. This in turn, might lead to less facial and nonverbal feedback and ultimately to higher extraneous cognitive load and also to higher fatigue. If learners only had to focus on a single camera view of a single person, extraneous cognitive load and fatigue would be reduced. The freed-up resources could be invested in germane processing. Therefore, we assume:

**Hypothesis 2:** Focus-view in videoconferences will lead to lower levels of fatigue compared to grid-view. In addition, we assume reduced extraneous and increased germane cognitive load when using focus-view.

As suggested by Wiederhold ^7^ and Peper and Yang ^25^, another method to combat videoconference fatigue might be to use plain backgrounds. Fosslien and Duffy^2^ argue that reducing on-screen stimuli could also result in less extraneous cognitive load [see also^26^]. From the field of organizational psychology, it is known that backgrounds in technology-mediated interviews can lead to distraction and biases in interpersonal evaluation^27–29^. Many videoconference programs offer the possibility to set up virtual backgrounds that can vary in color and complexity. However, colorful and complex backgrounds might increase extraneous cognitive load. Additionally, blurring effects might distract from the relevant content of the videoconference, further increasing extraneous cognitive load. In contrast, using a natural background could allow participants to focus more easily on relevant content, potentially reducing fatigue. The cognitive resources freed up could be invested in germane processing, thereby increasing germane cognitive load. Another argument for an increased investment of germane resources is that a more personal background in the natural view might foster greater interest in what the counterpart is saying. Accordingly, we assume:

**Hypothesis 3:** The use of natural backgrounds in videoconferences will result in lower levels of fatigue compared to when virtual backgrounds are used. Additionally, we anticipate a reduction in extraneous cognitive load and an increase in germane cognitive load when natural backgrounds are used.

Another strategy to reduce Zoom fatigue does not aim to alleviate unnecessary cognitive load, but rather focuses on promoting active engagement from learners. In addition to the technical layout of the conference, the didactic format of the conference content could also influence learners’ perceived load and fatigue. Videoconferences are often used primarily as a means of conveying information and thus tend to be more teacher-centered and less student-centered. To prevent fatigue, it might be beneficial for online educators to involve students more actively in the lessons^11^. Accordingly, Toney, et al.^30^ found in a rather exploratory study that switching activities, working in smaller online groups, and utilizing asynchronous teaching formats are effective strategies. This is further substantiated by a study from de Araujo Guerra Grangeia, et al.^31^ finding that actively involving learners through quizzes, surveys, and problem-based learning on an internet platform can increase learning success and reduce cognitive load. Furthermore, the findings of a qualitative study by Prasetyo, et al.^32^ suggest using collaboration and communication between lecturers and learners and more inclusive lessons in cyber education to overcome fatigue and pressure. Bennett, et al.^1^ also suggest that individuals feel less fatigued when they participate more actively, such as by unmuting themselves, participating in polls, or providing nonverbal feedback. Furthermore, Peper and Yang^25^ found that active participation in class can lead to more energy and less distraction, which could in turn also be associated with lower fatigue. In terms of the concepts mentioned above, less distraction would equate to less extraneous cognitive load, and more energy would result in increased germane cognitive load. However, none of these studies have provided empirical evidence supporting these strategies. To investigate whether increased student participation leads to less fatigue, we propose:

**Hypothesis 4:** Active student participation in videoconferences will result in lower levels of fatigue compared to scenarios with no active participation. Additionally, we anticipate a reduction in extraneous cognitive load and an increase in germane cognitive load when students actively participate in class.

## Method

This study was exempt from an ethic committee approval due to the recommendations of the German Research Association: All subjects were in no risk out of physical or emotional pressure, we fully informed all subjects about the goals and process of this study and none of the subjects were patients, minors or persons with disabilities. Participation was voluntary and all subjects signed a written informed consent and were aware that they had the chance to withdraw their data at any point of the study. The corresponding information can be found via: https://www.dfg.de/en/research-funding/proposal-funding-process/faq/humanities-social-sciences#263154

### Sample

To determine the required sample size to test our research questions with a power of 0.80, we conducted a power analysis using G*Power^33^. This analysis revealed a required sample size of *N* = 27 for a paired t-test to detect an intermediate-sized effect (*d* = 0.50).The initial sample consisted of 39 students (30 females, 9 males, age: *M* = 23.10, *SD* = 3.89) in a psychology bachelor’s program at a small German university. Participants reported spending an average of 23.86 h on average on their studies (*SD* = 12.17). Additionally, 59% had a paid part-time job (weekly working time *M* = 10.64 h, *SD* = 5.85) in addition to their studies. The sample consisted of the participants of two different seminars (*n* = 19, *n* = 21) with two different lecturers. One of the students attended in both seminars. The seminars were different in content (project management vs. digital transformation in industrial and organizational psychology), but in both seminars there was generally a high degree of interactivity and student-centered teaching. The participants of the seminars did not differ in age, weekly working time, or time spent on studying, all *t*s < 0.84, all *p*s  >0.41. However, the courses differed regarding their sex ratio, *t*(36) = 3.14, *p* = 0.003, with more female than male participants. Regarding technical equipment, 95% indicated that they were attending the online courses with a laptop/notebook. 15% indicated that they were additionally using either a desktop PC or a smartphone (multiple answers were possible). As Internet browser, students either used Mozilla Firefox (36%), Google Chrome (62%), Microsoft Edge (18%), or Safari (21%). Additionally, nine students indicated that they used two monitors.

### Design and procedure

Data collection took place at weekly intervals over an entire semester. Classes were held at the same time each week using the Zoom videoconferencing program (www.zoom.com). At the beginning of the semester, all participants were introduced to the research project and were asked to participate in a pre-test questionnaire, which asked about the demographic variables and participants’ technical equipment. Although the data collection was part of the seminar, participation in the research project was voluntary. Therefore, there was no incentive to participate in the study and there were no disadvantages to students for not participating in the study.

In the following weeks, the interventions followed in a weekly rhythm intervention vs. no intervention and thus, a weekly within-subjects design and not a fully crossed design. The different interventions were, self-view vs. no self-view, focus-view vs. grid-view, virtual background vs. natural background, and interaction vs. no interaction. In other words, the procedure could also be seen as four separate within-subjects studies. At the beginning of each session, participants were asked to set up their Zoom settings according to the intervention. For this purpose, an overview slide was shown in each case, explaining how to set up virtual backgrounds, for example. Participants were not blinded about the interventions except for the invention interaction vs. no interaction, for which no setup was needed. Each lesson lasted between 60 and 90 min and differed in their content just as in a normal weekly seminar. Directly at the end of the lesson, the students were asked to complete an online questionnaire. Each participant was asked to create an individual code, so that data collection was anonymous but we could match the pre-questionnaire and the weekly questionnaire. The weekly questionnaire contained questions on cognitive load (extraneous, germane, and intrinsic), distractions during the lessons (e.g. simultaneous use of the smartphone, social media, writing emails, etc.), and on fatigue. Intrinsic load was used as control variable to ensure that the difficulty of the learning material was comparable for the respective comparisons.

In order to increase the power and to eliminate the influence of individual differences and in the content of the seminars, we used a within-person design. However, since there is no compulsory attendance at German universities, the number of participants varied from week to week.

### Measures

#### Cognitive load

We used the Cognitive Load Questionnaire from Klepsch, et al.^34^ to measure cognitive load. The items contained two items on intrinsic cognitive load (e.g., “During the videoconference, many things needed to be kept in mind simultaneously”, α = 0.87), two items on germane cognitive load (e.g., “My point during the videoconference was to understand everything correct”, α = 0.87), and three items on extraneous cognitive load (e.g., “During the videoconference, it was difficult to recognize and link the crucial information”, α = 0.87) that should be answered on a 7-point rating scale ranging from 1 = strongly disagree to 7 = strongly agree.

#### Fatigue

Fatigue was measured with an adapted short German-language questionnaire from Martin, et al.^35^. It contained 11 items (e.g., “It was difficult for me to concentrate”) that should be answered on a 7-point rating scale ranging from 1 = *strongly disagree* to 7 = *strongly agree.* Coefficient alpha varied was α = 0.87.

### Interventions

#### Self-view vs. no self-view

The first intervention consisted of the activation of the self-view vs. the conscious deactivation of the self-view. As mentioned earlier, at the beginning of the seminar, students were asked to activate or deactivate the self-view. An instructional slide was used to show how this works.

#### Focus-view vs. grid-view

The second intervention consisted of the focus-view vs. grid-view setting. For both settings, students were shown a slide at the beginning of the intervention explaining how to set this.

#### Virtual background on vs. natural background

The third intervention was to turn on a virtual background. Students were free to choose a specific background. Blurring, however, was excluded. The lecturers used a background of the university’s corporate design. In the week without intervention, the natural background was used.

#### Active participation vs. no active participation

The fourth intervention was to interact with the students more often during the seminar by asking questions, using online tools with them, taking surveys, etc. In the week without active participation, the seminar consisted only of frontal teaching without interaction, i.e. no surveys or comprehension questions were used.

## Results

In all interventions, we used intrinsic cognitive load as a control variable to control if the content of the respective lessons were comparable with regard to complexity of the learning material. Like already mentioned, there is no compulsory attendance at German universities thus resulting in a varied number of participants from week to week. Accordingly, the differences in the degrees of freedom in the following sections can be explained by the fact that the attendance of the students for the respective comparisons of the weeks with an intervention vs. no intervention varied over the course of the semester. Only those students who were present in both sessions of a comparison (intervention vs. no intervention) were included in the data analysis. The means, standard deviations, and effect sizes for the different interventions are shown in Table 1,2,3,4.

**Table 1:** Means, Standard Deviations, and Effect Sizes for the Dependent Variables for the Self-View Intervention (n = 25).

	Self-view off		Self-view on		Cohen’s* d*
	*M*	(*SD*)	*M*	(*SD*)	
Intrinsic CL	2.86	(1.19)	3.86	(1.32)	-0.80**
Germane CL	5.50	(1.05)	5.28	(0.87)	0.25
Extraneous CL	1.64	(0.54)	2.52	(1.22)	-0.82**
Fatigue	2.25	(1.08)	3.41	(1.11)	-0.71**

CL = cognitive load.

* *p* < .05, ** *p* < .01.

**Table 2:** Means, Standard Deviations, and Effect Sizes for the Dependent Variables for Focus-View vs. Grid-View Intervention (n = 12).

	Focus-View		Grid-View		Cohen’s* d*
	*M*	(*SD*)	*M*	(*SD*)	
Intrinsic CL	3.54	(1.80)	3.38	(1.46)	0.08
Germane CL	5.38	(1.09)	5.17	(1.35)	0.27
Extraneous CL	2.44	(1.86)	2.22	(1.24)	0.18
Fatigue	2.52	(1.58)	3.18	(1.22)	-0.46

CL = cognitive load.

* *p* < .05, ** *p* < .01.

**Table 3:** Means, Standard Deviations, and Effect Sizes for the Dependent Variables for the Virtual Background Intervention (n = 14).

	Virtual Background on		Virtual Background off		Cohen’s* d*
	*M*	(*SD*)	*M*	(*SD*)	
Intrinsic CL	4.43	(1.41)	3.61	(1.30)	0.53*
Germane CL	4.43	(1.48)	5.61	(0.92)	-0.68*
Extraneous CL	3.14	(1.36)	2.45	(0.65)	0.59*
Fatigue	3.42	(1.39)	3.02	(0.98)	0.31

CL = cognitive load.

* *p* < .05, ** *p* < .01.

**Table 4:** Means, Standard Deviations, and Effect Sizes for the Dependent Variables for the Interaction Intervention (n = 28).

	Interaction		No interaction		Cohen’s* d*
	*M*	(*SD*)	*M*	(*SD*)	
Intrinsic CL	3.52	(1.52)	3.75	(1.25)	-0.13
Germane CL	5.43	(1.18)	5.61	(0.96)	-0.17
Extraneous CL	2.14	(1.02)	2.71	(1.38)	-0.40*
Fatigue	3.06	(1.03)	3.58	(1.21)	-0.39*

CL = cognitive load.

* *p* < .05, ** *p* < .01.

### Self-view vs. no self-view

The first intervention compared the effects of the self-view function regarding on fatigue and cognitive load. Therefore, we conducted a t-test for paired samples. The results on fatigue show that there is a significant difference, *t*(24) = 3.54, *p* < 0.001,* d* = 0.71. That means that deactivating self-view can go along with decreased fatigue. Furthermore, we found a significant difference for extraneous cognitive load, *t*(24) = 4.09, *p* < 0.001, *d* = 0.82, with higher values for the condition with the self-view on, but no significant difference for germane cognitive load, *t*(24) = 1.25, *p* = 0.11, *d* = 0.25. Furthermore, we found a significant difference for intrinsic cognitive load, *t*(24) = 4.00, *p* < 0.001, *d* = 0.80. Hypothesis 1 was, therefore, largely supported.

### Focus-view vs. grid-view

The second intervention compared the effects of the focus-view function vs. the grid-view function regarding cognitive load and fatigue. Therefore, we conducted a t-test for paired samples. The results on fatigue failed to reach significance, *t*(11) = 1.60, *p* = 0.07, *d* = 0.46. Moreover, none of the comparisons regarding the subscales of cognitive load reached significance, all *t*s < 0.93, all *p*s > 0.18, all *d*s < 0.28. Hypothesis 2 was, therefore, not supported.

### Virtual background on vs. natural background

The third intervention compared the effects of activating a virtual background vs. the natural background regarding cognitive load and fatigue. Therefore, we conducted a t-test for paired samples. The results on fatigue failed to reach significance, *t*(13) = 1.15, *p* = 0.14, *d* = 0.31. Hypothesis 3 was, therefore, not fully supported. However, we found significant differences for extraneous cognitive load, *t*(13) = 2.20, *p* = 0.02, *d* = 0.59, with higher values for the condition with the virtual background, and furthermore a significant difference for germane cognitive load, *t*(13) = 2.57, *p* = 0.01, *d* = 0.68 with higher values for the condition with natural background. Furthermore, we found a significant difference for intrinsic cognitive load, *t*(13) = 1.98, *p* = 0.04, *d* = 0.53.

### Active participation vs. no active participation

The last intervention compared the effects of interactions during classes vs. no interaction regarding cognitive load and fatigue. Therefore, we conducted a t-test for paired samples. The results on fatigue showed a significant difference, *t*(27) = 2.08, *p* = 0.02, *d* = 0.39. That means that interacting with students can go along with decreased fatigue. Furthermore, we found a significant difference for extraneous cognitive load, *t*(27) = 2.12, *p* = 0.02, *d* = 0.40, with higher values for the condition with no active participation. No significant effects were found for germane cognitive load, *t*(27) = 0.90, *p* = 0.19, *d* = 0.17, and intrinsic cognitive load, *t*(27) = 0.71, *p* = 0.24, *d* = 0.14. Hypothesis 4 was, therefore, largely supported.

## Discussion

The present study aimed to investigate the effectiveness of different interventions in reducing videoconference fatigue in university teaching during the Covid-19 pandemic. The interventions tested were self-view vs. no self-view, focus-view vs. grid-view, virtual background vs. natural background, and active participation vs. no active participation. The results provided valuable insights into the potential effectiveness of these interventions and shed light on their impact on fatigue and cognitive load. One of the strengths of our study is the within-subject design which allowed us to control for several factors, for example that social isolation during the Covid-19-pandemic could lead to general fatigue or depressive mood, which has been a limitation of several other studies [e.g., ^6^].

The findings of the study supported some of the hypotheses while also revealing unexpected outcomes. Switching off the self-view during videoconferences was found to significantly reduce fatigue, supporting Hypothesis 1. This finding is consistent with previous theoretical suggestions that the constant self-observation and the feeling of being watched can be exhausting^22,36^. It is important to note that deactivating the self-view not only reduced fatigue but also resulted in lower extraneous cognitive load. On the one hand, lower scores for extraneous cognitive load can be seen as further empirical evidence for a beneficial effect of switching off self-view. This suggests that the videoconference was less resource draining when participants were not constantly monitoring their own video feed. On the other hand, we also found lower values for intrinsic cognitive load, which could be due to a non-comparable complexity of the learning content in the weeks with self-view on vs. self-view off, limiting the generalizability of this finding.

Contrary to Hypothesis 2, no significant difference was found between focus-view and grid-view in terms of fatigue and cognitive load. This unexpected result could be attributed to the specific characteristics of the sample or the nature of the seminars. It is possible that the high level of interactivity and student-centered teaching as the standard approach in both seminars minimized the negative effects of the grid-view, as participants were actively engaged and received individual attention from the instructors. Further research with different samples and teaching formats is needed to explore the impact of focus-view and grid-view on videoconference fatigue.

Regarding the use of virtual backgrounds, the results did not support Hypothesis 3 regarding our assumptions about fatigue. There was no significant difference in fatigue between the virtual background and natural background conditions. However, it is worth noting that participants reported higher extraneous cognitive load when using virtual backgrounds compared to the natural background condition. This suggests that virtual backgrounds may introduce additional cognitive demands and distractions. Additionally, and in line with our hypothesis, the use of natural backgrounds leads to higher germane cognitive load. This means that the saved cognitive resources by using natural backgrounds can be used more efficiently for understanding the task. However, again significant differences in intrinsic cognitive load could be due to a non-comparable complexity of the learning content further limiting the generalizability of this result.

In support of Hypothesis 4, it was found that active student participation during videoconferencing significantly reduced fatigue. This finding is consistent with previous studies suggesting that active engagement and participation can enhance learning experiences and reduce cognitive load^1,3,25,30^. Involving students in polls, nonverbal feedback, and active discussion might have provided a sense of control and increased interest, thereby reducing fatigue. Furthermore, it is important to note that there was also a significant difference in extraneous cognitive load across the active participation and no active participation conditions. This suggests that the effects of active participation might be both specific to fatigue reduction and reducing extraneous cognitive load experienced by participants. However, it is noteworthy that participants did not report higher levels of germane cognitive load.

Overall, the present study contributes to the understanding of videoconference fatigue and provides empirical evidence for interventions in an actual teaching context that can help mitigate its effects. The findings highlight the importance of considering both the technological aspects (e.g., self-view, background settings) and pedagogical approaches (e.g., active participation) in online teaching to promote a more engaging and less fatiguing learning environment.

### Limitations and lines for future research

Besides the benefits of the findings of this study, it is important to acknowledge the limitations, including the small sample size and the specific context of the Psychology bachelor programme at a German university.

As already mentioned, one of the limitations of our study is the small sample size and thus reduced power. Some effects, such as the difference in fatigue between focus-view and grid-view, show promising effect sizes, but fell short of significance due to the small sample size. Although it is a great strength that the interventions were studied in the field, it would still be desirable to study the results in larger or more courses. However, examining the effects in more courses would also lead to more potential influencing factors like differences between lecturers, duration of classes [see also^3,12^], complexity of learning material etc. A first indication of this problem could be seen in the significant difference in intrinsic cognitive load between the teaching units in two of the interventions. Although we deliberately chose our study design, a between-subjects design in parallel courses could also be an idea for further research, where interventions that are assumed to have a positive effect are varied from week to week between the courses. In this way, learning could still be ensured despite testing different interventions. Additionally, learning success could be measured as a further dependent variable.

In addition, since attending classes is not compulsory at German universities, attendance continued to decline throughout the semester, further reducing the power of the later interventions (e.g., focus-view vs. grid-view). Testing our interventions in the field was also accompanied by the fact that we were unable to completely blind the students by announcing the intervention at the beginning of the courses. Although we did not inform the students of any prior assumptions about the effects of different interventions, it cannot be excluded that the students already made their own prior assumptions about the intervention by the announcement of an intervention, which could influence the responses. Additionally, we could not guarantee that, although we made the announcement at the beginning of the course, the students would actually use the settings. Additional studies could also investigate whether the electronic device used has an effect on the results. Research has found that working on smaller screens can lead to a greater strain on working memory^37,38^. In our study, 95% of participants stated that they attended the course with a laptop/notebook. However, in online teaching it is of course also possible to participate with devices that have a smaller screen (e.g., a smartphone).

Although we have already examined four possible interventions in the course of our study, this does not mean that all possible interventions have already been exhausted. Accordingly, further technical innovations also offer various possibilities that can potentially influence effort. For example, a relatively new setting in videoconferencing is the presentation in a fictitious meeting room, where the video windows are only shown onto a portrait in a fictitious meeting room or lecture hall or even using avatars within a virtual meeting room with additional options for communication and interacting. Another possible intervention could also be to not only hide one’s own video, but also to switch it off completely. In addition, there is of course the possibility of combining the interventions we tested with each other, which could reinforce certain positive, but also negative interaction effects. However, as a first step, the present study provides a promising approach to looking at the intervention individually.

Future research should investigate the extent to which various new technical solutions can be applied to reducing videoconference fatigue. As proposed by Peper, et al.^6^, camera considerations to enhance your media presence might also be part of future interventions. Considering media richness theory^4^ and findings concerning the effect of eye contact in videoconferences^39,40^, the position of your camera and, therefore, the immediacy of eye contact could also be a critical factor in improving communication and ultimately reducing fatigue^36^.

### Practical implications

In view of our results, it is definitely recommended for teachers, students, and other people in videoconference meetings to switch off the self-view in videoconferences. As our results show, this can reduce perceived fatigue. Another effect we could show in our study was that interactions reduced perceived fatigue, which is also in line with prior assumptions based on qualitative results from surveys ^6^. Therefore, it is also recommended for online teaching to interact with students, ask questions, take polls, etc.

One aspect mentioned by Wicks^10^ is that during the outbreak of the Covid-19 pandemic, teachers partly used the same lessons and lectures and just changed the medium – which of course could not be changed from one day to the other. However, and although only being partly based on our results, we encourage teachers and lecturers to adapt their lessons to online education facing the challenges that it is accompanied with. Accordingly, it seems reasonable to reduce screen time and limit the number of meetings, as this has been shown to positively influence well-being^3,12^.

## Conclusion

In conclusion, as online teaching and videoconferencing continue to play a significant role in education and business even after the Covid-19 pandemic is over, it is essential to develop evidence-based strategies to combat videoconference fatigue. The interventions tested in this study provide initial insights into potential approaches to reducing fatigue and improving the learning experience in online settings. By implementing interventions such as deactivating self-view, promoting active participation, and considering the use of natural backgrounds, educators, instructional designers, and also managers can improve the quality of online teaching and videoconference meetings and alleviate the negative effects of videoconference fatigue and cognitive load. Further research and ongoing exploration in this area is essential to continuously improve the effectiveness and well-being of participants in online learning environments.

## Data Availability

The data that support the findings of this study are available from the corresponding author upon reasonable request.
